# Effect of nutritional calcium and phosphate loading on calciprotein particle kinetics in adults with normal and impaired kidney function

**DOI:** 10.1038/s41598-022-11065-3

**Published:** 2022-05-05

**Authors:** Mark K. Tiong, Michael M. X. Cai, Nigel D. Toussaint, Sven-Jean Tan, Andreas Pasch, Edward R. Smith

**Affiliations:** 1 Department of Nephrology, The Royal Melbourne Hospital, Grattan Street, Parkville, VIC 3052 Australia; 2 Department of Medicine (RMH), University of Melbourne, Parkville, Australia; 3 Calciscon AG, Biel, Switzerland; 4 Lindenhofspital Bern, Bern, Switzerland; 5 Department of Physiology and Pathophysiology, Johannes Kepler University, Linz, Austria

**Keywords:** Chronic kidney disease, Phosphorus metabolism disorders

## Abstract

Plasma approaches metastability with respect to its calcium and phosphate content, with only minor perturbations in ionic activity needed to sustain crystal growth once nucleated. Physiologically, calcium and phosphate are intermittently absorbed from the diet each day, yet plasma concentrations of these ions deviate minimally post-prandially. This implies the existence of a blood-borne mineral buffer system to sequester calcium phosphates and minimise the risk of deposition in the soft tissues. Calciprotein particles (CPP), endogenous mineral-protein colloids containing the plasma protein fetuin-A, may fulfill this function but definitive evidence linking dietary mineral loading with their formation is lacking. Here we demonstrate that CPP are formed as a normal physiological response to feeding in healthy adults and that this occurs despite minimal change in conventional serum mineral markers. Further, in individuals with Chronic Kidney Disease (CKD), in whom mineral handling is impaired, we show that both fasting and post-prandial levels of CPP precursors are markedly augmented and strongly inversely correlated with kidney function. This study highlights the important, but often neglected, contribution of colloidal biochemistry to mineral homeostasis and provides novel insight into the dysregulation of mineral metabolism in CKD.

## Introduction

Calcium and phosphate form highly insoluble salts in aqueous solution and while metabolism in all living organisms is dependent on these essential nutrients, their coexistence in biological fluids creates an inherent mineralisation risk and the need for tight regulation^1^. Most extracellular fluids like plasma, are considered metastable or approaching metastability with respect to their calcium and phosphate ionic activities^2^, readily sustaining crystal growth if nucleated. This tendency for calcium and phosphate to precipitate has been harnessed by vertebrates for its unique biomechanical properties during the evolution of the skeletal and dental tissues. However, at the same time, this mandated mechanisms of rapid mobilisation and efficient bulk transport of mineral precursors to meet the demands of growth and repair of these hard tissues, as well as strategies of restricting mineralisation to these sites and preventing unwanted calcification in soft tissues^3^. Indeed, even modest elevations in plasma calcium and phosphate concentrations are associated with increased risk of pathological calcification of the arteries and soft tissues^4^. Since excess calcium and phosphate are almost exclusively excreted by the kidney, this risk, along with its associated cardiovascular sequelae, is substantially higher in those with Chronic Kidney Disease (CKD), where disturbances in mineral metabolism become evident with relatively small decrements in glomerular filtration rate (GFR)^5,6^. Given the abundance of calcium and phosphate in the diet to which mammals are intermittently exposed, one might suspect the risk of ectopic mineralisation to be highest following a meal due to the anticipated surge in mineral ions absorbed from the intestine. Yet, contrary to this expectation, plasma calcium and phosphate concentrations change minimally in the post-prandial period and while increased urinary excretion helps to maintain homeostasis^7^, this response is not instantaneous and lags by some hours after ingestion of millimolar amounts of calcium and phosphate^8^. As a solution to this physiological challenge, a blood-borne system for buffering and transporting mineral has long been suspected^9^, and recent attention has turned to colloidal mineral-protein complexes that could fulfill this function and safely chaperone the intermittent flux of mineral through extracellular fluid to sites of utilisation or disposal^10^.

In plasma, the most potent and abundant protein mineralisation regulator is the liver-derived glycoprotein fetuin-A^11^, which interacts with nascent colloidal mineral-protein complexes to form calciprotein particles (CPP)^12,13^. Analogous to the manner in which apolipoproteins solubilise their lipid cargo for transport, fetuin-A stabilises poorly soluble calcium phosphates, preventing crystal growth and precipitation, while facilitating their uptake in tissues for utilisation or clearance^14–16^. An inability to make or sufficiently stabilise CPP, as seen in fetuin-A knockout mice, results in one of the most severe phenotypes of ectopic calcification known^17^, where mineral-containing complexes precipitate directly in the lumen of the microvasculature leading to occlusion, ischaemia, necrosis and fibrosis^18^. CPP are generated through a series of ordered steps, initially with the binding of spontaneously formed clusters of calcium and phosphate ions to fetuin-A, forming calciprotein monomers (CPM)^19^, which then serve as the building blocks for larger polymeric structures; first, coalescing to form spherical primary CPP (CPP-I) containing amorphous calcium phosphate, before transforming into larger and denser ellipsoidal secondary CPP (CPP-II) containing crystalline hydroxyapatite^12^.

Beyond the purported physiological role of CPP formation in sequestering and dispersing excess mineral, elevated levels are also observed in states of impaired mineral metabolism, including in CKD^20^. In this setting, higher serum levels of CPM and CPP have been linked to an increased risk of cardiovascular events^21–25^ and mortality^22^. Pre-clinical studies suggest that CPP may directly induce vascular smooth muscle cell calcification^26,27^, activation of cellular inflammatory and cytotoxic pathways^28,29^, as well as vascular luminal and endothelial lesions^30^. Taken together, it has been proposed that prolonged exposure to chronically elevated levels of serum CPP may help explain the links between excess dietary mineral and poor patient outcomes in CKD^14,31^. Intriguingly, recent data also suggests that circulating CPM are filtered at the glomerulus^29^, implying that kidney impairment may affect CPP metabolism via multiple mechanisms.

Recent methodological advancements permit direct quantification of CPM^32^, CPP-I and CPP-II^33^, with these assays now applied across a number of observational and interventional clinical studies^24,25,34–39^. Another novel complementary method for assessing this system is the T50 test, a functional assessment of the capacity of serum to resist ex vivo formation of CPP-II, when challenged with supersaturating amounts of calcium and phosphate^40^. A lower T50 has been consistently associated with increased risk of vascular pathology and mortality in individuals with normal kidney function^41^, as well as in various CKD cohorts, including both non-dialysis^22,42,43^, and also dialysis-dependent CKD^44^. In addition to the kinetics of CPP-II formation, the size (hydrodynamic radius) of the CPP-II molecules generated in the T50 assay can also be measured^40,45^, and may provide additional prognostic information about vascular risk^46,47^.

The notion of a dietary origin of CPP is strongly supported by the observation that high-phosphate feeding is associated with increased ambient levels of CPM^48^ and CPP^33^ in animal studies, and the reduction in serum CPM^34^ and CPP^37^ in haemodialysis patients treated with intestinal phosphate binders. However, definitive observation of the effect of nutritional mineral intake on acute CPP kinetics in humans is lacking. Furthermore, the impact of CKD on post-prandial CPP metabolism has yet to be documented. To address these key evidence gaps, we conducted a controlled study of the effect of standardised food intake on serum CPM, CPP-I, CPP-II, T50 and CPP-II size, in fasted adults with normal or impaired kidney function.

## Methodology

### Study population

We studied 14 individuals with CKD and 16 age- and gender-matched healthy controls. Each participant had to be at least 18 years of age to be eligible. Participants were excluded if they: (i) had a history of mineral or bone disease, other than related to CKD; (ii) were being treated with an intestinal phosphate binder or calcitriol; or (iii) had a gastrointestinal disorder, history of lactose intolerance or were unwilling to consume the study meal. For the CKD group, we recruited seven individuals with an estimated glomerular filtration rate (eGFR) between 30 and 60 mL/min/1.73 m^2^ and seven with an eGFR < 30 mL/min/1.73 m^2^, excluding participants who required dialysis or with a previous kidney transplant. Healthy controls had no history of chronic medical conditions and had normal kidney function (eGFR > 60 mL/min/1.73 m^2^). The study was conducted in accordance with the Declaration of Helsinki.

All individuals provided written informed consent, and the study was approved by the local ethics committee (Melbourne Health Human Research Ethics Committee MH2018.363).

### Procedure

Each participant was studied after an overnight fast and sample collection commenced between 7.30 and 9.30 am. An intravenous cannula was inserted at the start of the study period. Before each blood sample was collected, an initial 5 mL draw from the cannula was discarded. Two initial fasting blood samples were taken 30 min apart to account for baseline variation. The mean values of these two timepoints were used as “time 0”. Immediately after collection of the second fasting sample, participants consumed a standardised meal (Sanitarium Up&Go liquid breakfast; 250 mL, vanilla flavour) containing 815 kJ energy, 300 mg calcium and 188 mg phosphate (Table 1). Participants were instructed to consume the entirety of the drink within 5 min. Serial blood samples were collected at five post-prandial timepoints (30, 60, 120, 180 and 240 min) from the commencement of the meal. During the study period participants were allowed to drink water but were not allowed to consume any other food or drink.

**Table 1:** Nutritional information for standardised meal (Sanitarium Up&Go liquid breakfast; 250 mL, vanilla flavour).

Parameter	Contents of meal^71^	Recommended dietary intake^a^
Energy (kJ)	815	–
Protein (g)	8.3	13%
Fat (g)	4.3	–
Carbohydrate (g)	28.4	–
Sodium (mg)	158	17–34%
Potassium (mg)	450	45%
Calcium (mg)	300	30%
Phosphate (mg)	188	19%

^a^Percentage of recommended dietary intake based on Australian adult male^72^ (where generalised recommendations are available).

### Outcome measures

Blood was collected for repeated measurement of novel markers of mineral metabolism (CPM, CPP-I, CPP-II, T50 and CPP-II size) at each timepoint. Fetuin-A was measured at each timepoint, given its role as the principal mineral-binding protein present in CPM and CPP. We also measured serum phosphate, total calcium, magnesium, albumin and bicarbonate at each timepoint, and serum intact parathyroid hormone (PTH) and intact fibroblast growth factor-23 (iFGF23) at three timepoints (0, 120 and 240 min). Serum citrate was measured at 0, 30 and 60 min. Serum urea, creatinine and 1,25 dihydroxyvitamin D were measured once at fasting baseline (0 min). Blood samples for novel markers of mineral metabolism, PTH, iFGF23, 1,25 dihydroxyvitamin D and serum citrate, were allowed to clot for 30 min before centrifugation, and then serum aliquots were stored at −80 °C until batch-analysis. All other biochemical measurements were performed at the time of sample collection using standard laboratory methods.

### Gel-filtration and flow cytometric assays for CPM and CPP

We employed two complementary assays to quantitate different fractions of the circulating CPP pool^49^. Both assays use the fluorescently-labelled bisphosphonate derivative, OsteoSense 680EX (Perkin Elmer), which binds specifically to solid-phase calcium phosphate and preferentially to crystalline phases (e.g. hydroxyapatite). The ‘gel-filtration’ method of Miura et al.^32^ was used to measure small (< 50 nm diameter), low-density mineral-laden fetuin-A colloids. Briefly, frozen serum samples were thawed for 24 h at 25 °C to induce aggregation of CPM and phase transition to crystalline calcium phosphate. Samples were then centrifuged for 30,000*g* for 2 h at 4 °C to remove larger CPP-I and CPP-II, leaving less dense crystal-laden fetuin-A monomer and multimers for staining with OsteoSense (0.5 µM) in HEPES-buffered DMEM (pH 8.0). Unbound dye was subsequently removed by gel filtration (Micro Bio-Spin^®^ Columns with Bio-Gel^®^ P-30, Bio-Rad) and the resultant fluorescence measured using an infrared scanner (Odyssey CLx, LI-COR; EX 685 nm, EM 700 nm). Miura and colleagues^32^ referred to the mineral detected as low-density (L)-CPP, however, here we refer to them as CPM to the reflect the origin of this mineral fraction in vivo. In our hands, the mean interassay analytical coefficient of variation (CV_A_) for the CPM assay was 4.9%.

For flow cytometric analysis, aliquots of frozen serum were processed using the standardised procedures described previously^50^. CPP-I and CPP-II were measured as previously described using a BD FACSVerse flow cytometer setup to resolve particles < 200 nm from background and operating with fluorescence triggering on OsteoSense-positive events^33,37^. In this assay, CPP are distinguished from membrane-delimited mineral-containing extracellular vesicles using phosphatidyl serine–binding lactadherin-FITC (Haematologic Technologies Inc., Essex Junction, VT). CPP-I and CPP-II were distinguished by differences in side scatter (SSC) intensity (related to particle size) and OsteoSense fluorescence intensity. Interassay CV_A_ for CPP-I and CPP-II were < 15% and < 10%, respectively.

### Other mineral markers

Serum T50 was measured by Calciscon AG, Biel, Switzerland, as previously described using a Nephelostar nephelometer (BMG Labtech, Ortenberg, Germany)^40^. The mean interassay CV_A_ for T50 was 3.4%. CPP-II hydrodynamic radius was measured by dynamic light scattering using a DynaPro Plate Reader II (Wyatt Technology, Santa Barbara, CA, USA) as described by Chen et al.^47^ The interassay CV_A_ for CPP-II size was 4%. Commercial immunoassays were used to measure iFGF23 (Kainos Laboratories, Tokyo, Japan), 1,25 dihydroxyvitamin D Immunodiagnostic Systems, Boldon, UK), and fetuin-A (R&D Systems, Minneapolis, USA) according to the manufacturer’s instructions. Mean interassay CV_A_ were 3.8%, 5.5%, and 3.2%, respectively. Serum citrate was measured using a colorimetric assay (Sigma-Aldrich, Darmstadt, Germany) with a mean interassay CV_A_ of 3.5%.

### Statistical analysis

Using GLIMMPSE, a validated linear mixed model power and sample size calculator^51^, we estimated that 10 participants would provide > 90% power to detect a doubling of CPP in the post-prandial period in with a type I error rate of 0.05. The study was not powered to detect a difference between groups.

Demographic and fasting biochemical data were compared between groups using unpaired t-test or Kruskal–Wallis test for normal and skewed continuous variables respectively, and chi-squared test for categorical variables.

Our aim was to describe the within and between-group post-prandial response for each repeated parameter. In order to do this, we fitted linear mixed-effects models (LMM) for each parameter, using a restricted maximum likelihood approach and with an unstructured covariance matrix^52^. For each LMM we modelled group, categorical time and group-by-time interaction as fixed effects and a random intercept was included for each participant to account for correlation of repeated measures. The control group and time ‘0’ were used as the reference values for group and time, respectively. Coefficient estimates for group-by-time interaction terms were used to test for differences in post-prandial response between the CKD and control group. After fitting each LMM, we also performed post hoc pairwise comparisons to test for differences in mean values between groups at each timepoint, and to test for deviation from the fasting baseline within each group. For these pairwise comparisons we used Bonferroni correction method to adjust for multiple comparisons. CPM, CPP-I, CPP-II, CPP-II size, PTH and iFGF23 were natural log transformed before fitting LMMs to ensure normal distribution of residuals. For ease of interpretation, coefficient estimates for interaction terms from these models were then exponentiated to derive estimates of percentage change. For the LMM of CPM, group by time interaction terms suggested a significant difference in post-prandial response between groups. To further explore the effect of kidney function on post-prandial levels of CPM we also calculated the area under the curve (AUC) for CPM using the cubic spline method (time 0 to 240 min), and examined the relationship between eGFR, AUC and maximum concentration of CPM (using Spearman rank correlation) as well as between CKD and time of maximum CPM concentration (using chi squared test).

Several samples had undetectable levels of CPP-I (2 samples in the control group, and 6 in the CKD group) or CPP-II (5 in the control group and 10 in the CKD group). For the main analyses, the lower limit of quantification for the assay (133 particles/mL) was used for these left-censored values. To assess for potential bias from this approach, we performed a sensitivity analysis where LMMs for CPP-I and CPP-II were refitted after imputing left-censored values using multi-level Tobit regression, where time and group were entered as independent variables^53^.

Two-tailed p values < 0.05 were considered significant. All data were analysed using Stata MP version 17.0 (StataCorp, College Station, USA) and figures were produced using GraphPad Prism version 9.2.0 (GraphPad Software, San Diego, USA).

## Results

### Participant demographics and clinical characteristics

The demographics and participant characteristics for each group are displayed in Table 2. Overall, the groups were well matched for age and sex. The mean eGFR within the CKD group was 29.2 mL/min/1.73 m^2^. Consistent with group allocation, participants within the CKD group had higher fasting levels of serum creatinine, urea, phosphate, PTH and iFGF23, and lower fasting eGFR, serum albumin, bicarbonate and 1,25-dihydroxyvitamin D. Fasting serum total calcium and magnesium were comparable between groups. Among the 14 CKD participants, one was hyperphosphataemic (> 1.50 mmol/L) and 10 (71%) were hyperparathyroid (> 10.0 pmol/L), while all participants in the control group had values within the respective population-based reference intervals for these two parameters. Medication use for the CKD group is shown in Supplementary Table S1.

**Table 2:** Participant demographics and clinical characteristics.

	Healthy control (n = 16)	CKD (n = 14)	p-value
**Participant characteristics**			
Age (years)	44.1 ± 13.5	44.3 ± 16.0	0.917
Female sex, n (%)	9 (56.3)	8 (57.1)	0.961
**CKD stage, n (%)**			
Stage 3a or stage 3b	–	7 (50.0)	
Stage 4 or stage 5 (non-dialysis)	–	7 (50.0)	
**Primary cause of kidney disease, n (%)**			
Diabetic nephropathy	–	2 (14.3)	
Glomerulonephritis	–	6 (42.9)	
Reflux nephropathy	–	2 (14.3)	
Polycystic kidney disease	–	4 (28.6)	
**Fasting biochemical parameters**			
Creatinine (µmol/L)	71.6 ± 9.9	239 ± 110.3	**0.001**
eGFR^a^ (mL/min/1.73 m^2^)	99.8 ± 10.2	29.2 ± 14.5	** < 0.001**
Urea (mmol/L)	4.8 ± 1.4	15.2 ± 7.4	** < 0.001**
Calcium (mmol/L)	2.29 ± 0.08	2.25 ± 0.10	0.262
Phosphate (mmol/L)	0.96 ± 0.14	1.17 ± 0.24	**0.006**
Magnesium (mmol/L)	0.84 ± 0.06	0.80 ± 0.08	0.132
Albumin (g/L)	40 ± 2.0	36 ± 2.9	** < 0.001**
Bicarbonate (mmol/L)	27 ± 2.6	22 ± 3.0	** < 0.001**
PTH (pmol/L)	5.2 (4.6–6.75)	16.9 (7.1–31.0)	**0.001**
1,25-dihydroxyvitamin D (pmol/L)	137 (103–173)	42 (31–56)	** < 0.001**
Intact FGF23 (pg/mL)	48.8 (32.9–68.8)	132.5 (75.1–238.9)	**0.002**

P-value is for between group difference, examined using unpaired t-test or Kruskal–Wallis test for normal and skewed continuous variables respectively, and chi-squared test for categorical variables.

Mean ± SD, number (percentage) or median (interquartile range).

*CKD* chronic kidney disease, *eGFR* estimated glomerular filtration rate, *FGF23* fibroblast growth factor-23, *PTH* intact parathyroid hormone.

^a^eGFR—calculated using CKD—Epidemiology Collaboration equation.

### Novel markers of mineral metabolism

**Figure 1:**
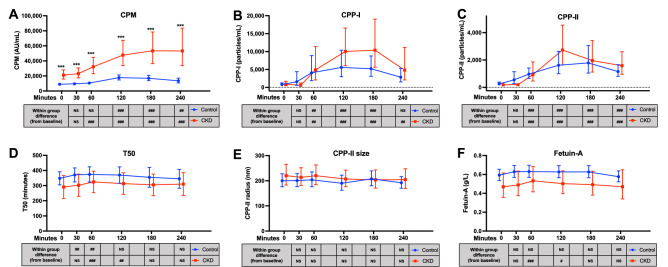
Post-prandial response of novel markers of mineral metabolism after standardised meal in participants with normal and impaired kidney function. Repeated measures of novel markers of mineral metabolism depicted for control and CKD group when fasting (“0”) and then post consumption of a standardised meal. Data are presented as mean and 95% confidence interval or geometric mean and 95% confidence interval. Data points have been offset for clarity. Between-group and within-group pairwise comparisons shown for each timepoint, with Bonferroni correction for multiple comparisons. **(A)** Calciprotein monomers (CPM); **(B)** primary calciprotein particles (CPP-I); **(C)** secondary calciprotein particles (CPP-II); **(D)** T50; **(E)** secondary calciprotein particle size (CPP-II size); **(F)** Fetuin-A. ***p < 0.001 for CKD group versus healthy controls. NS: p ≥ 0.05 versus group baseline; ##: p < 0.01 versus group baseline; ###: p < 0.001 versus group baseline.

Levels of novel markers of mineral metabolism at fasting and after the standardised meal for each group are depicted in Fig. 1. Summary data and group-by-time coefficient estimates are provided in Supplementary Table S2.

### CPM

Serum CPM was significantly higher in CKD participants than in controls when fasting, and at each post-prandial timepoint (Fig. 1A). A significant increase in serum CPM was observed post meal within each group. This increase began at 120 min post meal in the control group and 60 min post meal in the CKD group. Levels of CPM remained above fasting levels in both groups at the end of the study period (240 min).

Group-by-time interaction coefficients showed significant between-group differences for CPM at multiple timepoints, indicating that the post-prandial increase in CPM was proportionately larger in the CKD group than in controls (Supplementary Table S2). The peak difference between groups was seen at 240 min, where the increase from fasting levels was 73.3% higher in the CKD group than in controls (95% CI 30.8, 129.6, p < 0.001). To further explore the apparent effect of kidney function in post-prandial profile, CPM AUC, maximum concentration, and time of maximum concentration were also examined (Supplementary Fig. S1). There was strong, inverse correlation between eGFR and CPM AUC (r = −0.806, p < 0.001) and maximum CPM concentration (r = −0.779, p < 0.001). In addition, the time of maximum concentration for CPM occurred significantly later in the CKD group compared to controls.

### CPP-I and CPP-II

Significant post-prandial excursions were observed within both groups for CPP-I and for CPP-II (Fig. 1B,C). For CPP-I, serum levels were significantly increased from fasting baseline in both groups at 60 min. At the end of the observation period, CPP-I remained significantly higher than fasting values in the CKD group but were comparable to baseline in the control group. For CPP-II, significant post-prandial increases were evident in both groups at 60 min, and levels remained above fasting values at the end of the study period.

There was no significant difference between group means in pairwise comparisons at any timepoint for CPP-I or CPP-II (Fig. 1B,C). However, there was a significant group-by-time interaction for CPP-II at 120 min (+ 130.1% for the CKD group [95% CI 4.1, 408.6], p = 0.039) (Supplementary Table S2). Refitting LMMs for CPP-I and CPP-II after re-imputing left censored values using Tobit regression did not materially alter coefficient estimates, or post hoc between group or within group pairwise comparisons (Supplementary Table S3 and S4). In contrast to CPM, we found no association between eGFR and the AUC, maximal concentration or time to maximum concentration for serum CPP-I or CPP-II (Supplementary Fig. S2).

### T50, CPP-II size, fetuin-A and citrate

In both control and CKD groups, early but transient within-group increases in T50 from fasting levels were observed (Fig. 1D). This was evident at 30 and 60 min in the control group and at 60 and 120 min in the CKD group. There were no between-group pairwise differences, or significant group-by-time interactions for T50 at any timepoint. Within each group, CPP-II size appeared stable over the post-prandial period, with no significant excursions from baseline values in either group (Fig. 1E). There were no significant pairwise differences between groups at any timepoint, however at 180 min there was a modest, but significant, group-by-time interaction (−10.9% for CKD group [95% CI −19.5, −1.3]; p = 0.027). For serum fetuin-A, there were no significant pairwise group differences, or group by time interactions (Fig. 1F). However, in the CKD group, there was a transient post-prandial increase in fetuin-A, which was coincident with the increase in T50. In further ad hoc exploratory analysis of all participants, a strong correlation between deviation in T50 and fetuin-A from baseline was evident, irrespective of renal function (r = 0.839, p < 0.001; Supplementary Fig. S2). Since the study meal was noted to contain citrate, a known inhibitor of CPP formation and modulator of T50^54^, we measured serum citrate concentrations in available stored serum at baseline, 30, and 60 min. Although a small increase in serum citrate was observed in the CKD group at 30 min (Supplementary Table S3), this did not coincide with the observed changes in T50 in this group, nor was there any overall correlation between change in T50 and serum citrate (Supplementary Fig. S3).

### Conventional biochemical markers

**Figure 2:**
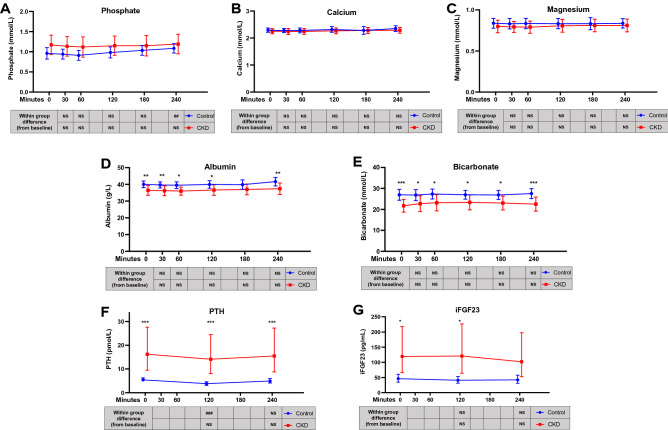
Post-prandial response of conventional markers of mineral metabolism after standardised meal in participants with normal and impaired kidney function. Repeated measures depicted for control and CKD group when fasting (“0”) and then post consumption of a standardised meal. Data are presented as mean and 95% confidence interval or geometric mean and 95% confidence interval. Data points have been offset for clarity. Between-group and within-group pairwise comparisons shown for each timepoint, with Bonferroni correction for multiple comparisons. **(A)** Phosphate; **(B)** calcium; **(C)** magnesium; **(D)** albumin; **(E)** bicarbonate; **(F)** intact parathyroid hormone (PTH); **(G)** intact fibroblast growth factor-23 (iFGF23). *p < 0.05 for CKD group versus healthy controls; **p < 0.01 for CKD group versus healthy controls; ***p < 0.001 for CKD group versus healthy controls. NS: p ≥ 0.05 versus group baseline; ##: p < 0.01 versus group baseline; ###: p < 0.001 versus group baseline.

Fasting and post-prandial levels of conventional biochemical markers are depicted in Fig. 2, and summary data and group-by-time coefficient estimates are also shown in Supplementary Table S4 and Supplementary Table S5.

There was a trend for serum phosphate to increase from fasting levels post meal in the control group, which reached significance at 240 min (Fig. 2A). By comparison, no within group post-prandial change was observed in the CKD group. This subtle difference in post-prandial response between groups was reflected in significant group-by-time interactions at 180 min (−0.09 mmol/L for CKD group [95% CI −0.17, −0.02], p = 0.018) and at 240 min (−0.10 mmol/L [95% CI −0.19, −0.02], p = 0.019). For serum calcium and magnesium, there were no significant between-group or within-group differences, nor significant group-by-time interactions (Fig. 2B,C, Supplementary Table S4). Serum albumin was lower in the CKD group compared to controls at all but one timepoint and remained unchanged from baseline levels in both groups post-prandially (Fig. 2D). Serum bicarbonate was higher in control than in CKD participants at all timepoints (Fig. 2E). There was no significant deviation from baseline in either group, however there was a significant group-by-time interaction at 120 min (+ 1.64 mmol/L for CKD group [95% CI 0.25, 3.04], p = 0.021). This coincided with the T50 peak in the CKD group, and in ad hoc analysis, there was correlation between the deviation in T50 and bicarbonate from baseline (Supplementary Fig. S4), albeit this was weak compared to the apparent interdependence of T50 and fetuin-A (Supplementary Fig. S2).

With respect to the major hormonal mineral regulators, serum PTH was significantly higher in the CKD group than in controls at all measured timepoints (Fig. 2F). There was a transient drop in PTH from fasting to 120 min in the control group, which was not seen in the CKD group. This corresponded to a significant group-by-time interaction at 120 min (+ 25.2% for CKD group [95% 5.5, 48.5], p = 0.01). Serum iFGF23 was significantly higher in the CKD than control group at baseline and 120 min, but not at 240 min (Fig. 2G). No significant post-prandial within group changes were observed.

## Discussion

To the best of our knowledge, this is the first study to report a significant post-prandial effect of nutritional intake on serum levels of CPM, CPP-I and CPP-II in humans. These effects were common to individuals with normal and impaired kidney function; however, the post-prandial excursion of serum CPM was much more pronounced in CKD participants. We also found an early and transient post-prandial effect on T50, which was present regardless of kidney function, and accompanied by a concomitant increase in serum fetuin-A.

Our findings are consistent with the notion that intestinal absorption of a dietary mineral load can directly lead to formation of circulating CPM, CPP-I and CPP-II. This has previously been proposed^10,15,16^, but based largely on animal data^33,48^. In contrast, evidence in humans has been indirect, coming from studies of intestinal phosphate binders in haemodialysis dependent CKD patients^34,37,55^. A small study by Yamada et al. suggested diurnal variation in serum CPP with post-prandial peaks, however this study used an older assay technique that was unable to separately quantify CPM and CPP sub-species, and participants were all hospitalised for management of unstable diabetes^56^. In contrast, all participants in this study were clinically stable, and as far as we are aware, for the first time we have demonstrated that post-prandial excursions are seen not only in individuals with CKD, but also in healthy adults, substantiating a role of CPM and CPP formation in the normal physiological response to the ingestion of food.

Surges of serum CPM and CPP were seen even after relatively modest, and physiologically relevant, nutritional mineral loads (Table 1). In contrast, there was minimal post-prandial variation in the more conventional markers of mineral metabolism. Previous studies have similarly shown limited post-prandial deviation in phosphate^7,57^, except when subjects are challenged with large pharmacological loads^58,59^. In health, total body phosphate is regulated, such that net intestinal absorption is matched by urinary excretion^8^. However, this response is not instantaneous, and a lag of several hours may be seen before augmentation of urinary phosphate excretion occurs, even when the phosphate load is given intravenously^8^. Instead, animal models have demonstrated that other, non-renal, mechanisms serve to maintain serum ionic concentrations more acutely, via distribution to bone and other tissues^59–61^. It is plausible that formation of CPM and CPP may be an important additional temporary depot of phosphate (and calcium), that is able to acutely buffer local mineral loads, such as from the gastrointestinal system^14^. The physiochemical properties of CPM and CPP mean that potentially large quantities of otherwise insoluble mineral can exist in the circulation without risk of precipitation, which ostensibly facilitates mineral to be safely transported in bulk to sites of use or clearance.

We observed a transient increase in serum fetuin-A in the CKD group. In contrast, fetuin-A appeared stable in controls, however, when pairwise comparisons were repeated without correction for multiple comparisons, there were significant increases at 30 and 60 min in controls (Supplementary Table S8), indicating that we may have been underpowered to detect an underlying effect. Beyond being a negative acute phase reactant, with levels strongly suppressed in response to acute and chronic inflammation^62^, little is known about other mechanisms that directly regulate the hepatic synthesis and secretion of fetuin-A, and there is a paucity of previous data about diurnal, or acute post-prandial variation in any species. Given the observed rise in serum fetuin-A after feeding, especially in the CKD group, and the requisite role of fetuin-A in CPM and CPP formation, it is intriguing to consider whether feeding may be “sensed” via a yet unknown mechanism, leading to hepatic release of fetuin-A to coincide with an influx of mineral from the intestines. Indeed, Uedono et al. recently suggested that CPP itself, may be a trigger for fetuin-A expression in cultured hepatocytes^63^. Thus, mechanisms controlling fetuin-A release and their response to mineral loading warrant further investigation.

T50 is a functional assessment of the serum’s ability to resist ex vivo CPP-II formation, representing a composite of various potentiating (including calcium and phosphate) and inhibiting (including fetuin-A, albumin, magnesium, citrate and bicarbonate) calcification factors^40^. We observed an early but transient increase in T50. This was seen in both groups, but in the CKD group, the peak was coincident with increases in serum fetuin-A. Further, among all participants, change in T50 from baseline was very closely correlated with change in serum fetuin-A (Supplementary Fig. S2), suggesting that fetuin-A may be the main factor underlying the observed changes in T50. Indeed, we did not find corresponding early post-prandial changes in other known modulators of T50 (serum phosphate, calcium, bicarbonate, magnesium, or albumin), although this does not exclude more subtle changes in one, or a combination, of these factors. For instance, a positive group-by-time interaction was present for serum bicarbonate in the CKD group at 120 min, which overlapped with the increase in T50. As for fetuin-A, there was also correlation between change in bicarbonate and T50 from baseline (Supplementary Fig. S4), albeit this was relatively weak compared to the correlation with the former (r = 0.385 vs. r = 0.839). Nevertheless, the post-prandial “alkaline tide” is a recognised phenomenon, and it is plausible that multiple factors contributed to the observed post-prandial changes in T50^64^. The study meal also contained a surprising amount of citrate (~ 16 mM), a known potent inhibitor of CPP formation, however, although there was a small increase in serum citrate in the CKD group, this change did not coincide with the observed changes in T50, nor was there any overall correlation between change in T50 and serum citrate across both groups. Other previously described modulators of T50, such as pyrophosphate and zinc, were not measured in this study. Although it is possible that these, or other yet unknown, factors may also have contributed to the observed post-prandial increase in T50, we note that quite substantial changes in concentration of these small inorganic molecules are generally required to significantly impact T50^65^. In contrast to the post-prandial effects on T50, the hydrodynamic radius of CPP-II remained ostensibly unchanged in both groups.

While post-prandial excursions of CPM were clearly observed in both groups, CPM levels were consistently higher in the CKD group at each of the timepoints, and group-by-time interaction terms indicated that the magnitude of the post-prandial response was significantly larger in CKD participants than in controls. Accordingly, in exploratory analysis, there was strong correlation between eGFR and CPM AUC and maximum concentration. Peak levels of CPM also tended to occur later in the CKD group. Theoretically, these differences could indicate an increased production of CPM following nutritional intake and/or an impaired capacity to clear CPM in those with CKD. The latter is supported by recent data by Koeppert et al., who used live two-photon microscopy to show that circulating CPM are predominantly cleared by glomerular filtration in mice^29^. A reasonable prediction therefore, may be that the capacity to clear CPM becomes impaired as GFR declines. However, alterations in other aspects of mineral homeostasis, including bone turnover^66^, and delayed renal excretion of acute phosphate loads^7,59^ are also commonly seen in CKD and may well have contributed to elevations in post-prandial levels. Recent evidence has questioned the long-held notion that CKD affects net intestinal phosphate absorption^57,67,68^. We did however note that six of the 14 individuals in the CKD group were taking cholecalciferol. Considering the potential for stimulatory effects of vitamin D on intestinal mineral absorption, we performed an ad hoc exploratory analysis to test whether use of nutritional vitamin D impacted the acute response of CPM, CPP-I and CPP-II to feeding (Supplementary Tables S9–S11). Apart from a single positive group-by-time interaction for CPP-I at 30 min in those taking cholecalciferol, all other interaction terms and post hoc pairwise comparisons for CPM, CPP-I and CPP-II were not significant, suggesting that cholecalciferol use was unlikely to have had a substantial effect on post-prandial CPM/CPP kinetics. While the small sample size and potential for confounding may preclude definitive conclusions, the lack of effect may also reflect the predominance of phosphate absorption via the paracellular pathway when mineral is abundant, which is not actively regulated by the vitamin D axis^69^.

In contrast to CPM, we did not observe a strong effect of kidney function on either CPP-I or CPP-II. Unlike CPM, circulating CPP is primarily cleared from the circulation by non-renal mechanisms. Animal and in vitro models have suggested rapid clearance of CPP-I predominantly by liver sinusoidal endothelial cells^28^, and of CPP-II by resident macrophages of the liver and spleen^70^. It is plausible that while participants in our CKD group displayed evidence of altered CPM metabolism, that these discrete CPP clearance pathways were sufficient to maintain normal post-prandial CPP profiles. We had anticipated that the CKD group would have discerningly higher levels of CPP compared to controls based on previous studies, albeit these studies enrolled patients with more advanced CKD who were dialysis dependent^33^, or used the older and indirect method of CPP measurement^20,23^. It is possible that individuals with more advanced CKD than those studied here may exhibit greater differences in fasting and post-prandial CPP levels. The lack of separation for CPP between our CKD and control group may also be due to our limited participant numbers, which also limited our ability to formally test for an effect of CKD stage (Supplementary Fig. S6). Indeed, our study was powered based on examining post-prandial responses rather than between group differences. Of note, CPP-I appeared to return to fasting levels earlier in controls than CKD participants, and there was a positive group-by-time interaction for CPP-II in CKD participants at 120 min. Both findings potentially signal that we may have observed more pronounced between-group effects in a larger cohort. Higher analytical imprecision for flow cytometry-based CPP measurements may have also contributed to the null findings.

In addition to providing new insights into the physiology of CPM and CPP metabolism, our study also has direct relevance for optimising the use of these novel markers of mineral metabolism in future studies. While each of these novel assays have shown promise in early clinical work, very few studies have reported^43^ or controlled for fasting/absorptive status^46,56^. In this study a number of individuals had undetectable levels of CPP-I or CPP-II in the fasting and early post-prandial period, and this profound effect of fasting on serum CPP is in itself a previously unreported and notable finding. In contrast, fasting CPM levels were measurable and significantly higher in the CKD group than in controls, which suggests that sustained elevations in CPM may not necessarily manifest in elevated CPP levels in those with non-dialysis-dependent CKD.

Previous epidemiological studies have suggested links between elevated levels of CPM and CPP with a range of surrogate markers of vascular disease^21,23,24^, as well as with cardiovascular events^25^ and all-cause mortality^22^. Further, lab-based studies have provided plausible mechanisms by which CPP may themselves mediate these pathological vascular outcomes^26–28^. However, while in vitro studies have suggested that many of these toxicities are induced in a dose-dependent manner, if CPP do have a normal physiological role in health then it is unclear at what point these particles may become injurious. Knowledge of the effect of nutritional intake on CPP kinetics as revealed here may prove crucial to further understanding this process. It is possible that a threshold concentration for the onset of pathological effects of CPM and CPP may (at least initially) only be reached in the post-prandial state in vivo. If so, dynamic testing of the post-prandial response may provide additional opportunities to evaluate earlier manifestations of dysregulated mineral metabolism, as well as the risk of associated vascular disease. Another important possibility is that the composition and thus the intrinsic toxicity of CPP-I and CPP-II differs among health and CKD^45^.

### Limitations

We acknowledge that this study has several limitations, including limited patient numbers, as already discussed. Notably, we also used a standardised meal given after an overnight fast, and so cannot comment on the effect of varying meal composition or the effect of subsequent meals given throughout the rest of the day. We chose the meal based on its commercial availability, allowing for standardisation between participants, and because it represented physiologically relevant nutritional loads (Table 1). It is however possible that if participants were challenged with larger mineral loads, that further separation between those with normal and impaired kidney function may have been apparent. Similarly, given we only observed individuals for four hours post-meal, it is conceivable that the cumulative effect of subsequent meals may have also distinguished groups further.

We did not recover faeces or urine and so cannot comment on total mineral absorbed or excreted during the study. We assume that changes in each measure seen after feeding are directly related to nutritional intake. This assumption is supported indirectly by detailed imaging studies showing flux of calcium and phosphate ions following food intake^57^, as well as direct evidence from animal studies showing that acute oral gavage of mice with a buffered phosphate solution results in serum spikes in CPM/CPP^48^. Nevertheless, we did not observe participants over extended fasting conditions, and so cannot conclusively account for non-dietary related diurnal fluctuations in any of the studied parameters. We did however have two fasting samples, the average of which was used as “time 0”, and variability between these fasting samples was trivial compared to the magnitude of changes seen in the post-prandial period (Supplementary Fig. S5).

We used the Bonferroni method to account for the multiple post hoc pairwise comparisons between and within groups for each parameter. This is undoubtedly a conservative approach, and to our knowledge not one adopted by no other similar feedings studies of post-prandial mineral metabolism. We chose this approach given the large number of timepoints and comparisons that were made and reasoned that it was preferable to focus on the most robust and significant signals. However, as a result we may have missed smaller, but potentially relevant effects. We did not correct for multiple testing across different mineral parameters given the likelihood of interdependent physiologically linked changes.

## Conclusion

Our study has revealed for the first time that nutritional mineral intake leads to the formation of CPM and CPP in blood as a normal physiological response to feeding. These findings corroborate the hypothesis that CPM/CPP formation helps to buffer post-prandial mineral loads, functioning as a temporary circulating store of bulk calcium phosphates ultimately destined for utilisation/storage (e.g., mineral precursor for bone mineralisation) or elimination. We also observed higher fasting levels of serum CPM and a larger post-prandial response in those with impaired kidney function, suggesting that CPM metabolism is manifestly altered in CKD. Analysis of post-prandial CPM/CPP handling may provide new insights into the mechanisms linking excessive dietary calcium and phosphate intake to increased risks cardiovascular disease in patients with impaired mineral excretion. More broadly, these novel findings underscore the important, but often neglected, contribution of colloidal biochemistry to mineral homeostasis.

## Supplementary Information

Supplementary Information.

## Data Availability

The data that support the findings of this study are available on request from the corresponding author. The data are not publicly available due to privacy or ethical restrictions.
