# Supplementary material for: Effect of nutritional calcium and phosphate loading on calciprotein particle kinetics in adults with normal and impaired kidney function
Source: Sci Rep. 2022 May 5;12:7358. doi: 10.1038/s41598-022-11065-3 (PMC9072391; doi:10.1038/s41598-022-11065-3)
Supplement: Supplementary file 1 — Supplementary Information. [file 41598_2022_11065_MOESM1_ESM.pdf]

Supplementary Material

Effect of nutritional calcium and phosphate loading on calciprotein particle kinetics in adults with normal and impaired kidney function

*Mark K. Tiong[1,2], Michael MX Cai,[1] Nigel D. Toussaint[1,2], Sven-Jean Tan[1,2], Andreas Pasch[3-5]and *Edward R. Smith[1,2]

[1]*Department of Nephrology, The Royal Melbourne Hospital, Parkville, Australia;*
[2]*Department of Medicine (RMH), University of Melbourne, Parkville, Australia;*
[3]*Calciscon AG, Biel, Switzerland;*
[4]*Lindenhofspital Bern, Bern, Switzerland;*
[5]*Department of Physiology and Pathophysiology, Johannes Kepler University, Linz, Austria.*

***Joint corresponding authors:**

Dr Mark K. Tiong
Department of Nephrology
The Royal Melbourne Hospital,
Grattan Street, Parkville, Victoria 3052,
Australia
Telephone +61 3 9342 7143
E-mail: mark.tiong@mh.org.au

Associate Professor Edward R. Smith
Department of Nephrology
The Royal Melbourne Hospital,
Grattan Street, Parkville, Victoria 3052,
Australia
Telephone +61 3 9342 3065
E-mail: edward.smith@mh.org.au

Supplementary Table S1: Medications for CKD group

Medication	Number taking
Calcium carbonate	1 (7.1)
Cholecalciferol	6 (42.9)
Prednisolone	4 (28.6)
Non-steroid immune suppressant	3 (21.4)
Diuretic	7 (50.0)
ACE-I, ARB or MRA	12 (85.7)
Other anti-hypertensive agent	5 (35.7)
Statin	4 (28.6)

Medications taken at time of study for CKD group. Healthy controls were not taking regular medications. Results are presented as number (percentage).

Abbreviations: ACE-I, angiotensin-converting enzyme inhibitor; ARB, angiotensin II receptor blocker; MRA, mineralocorticoid receptor antagonist.

Supplementary Table S2: Linear mixed-effect models for novel markers of mineral metabolism

	**CPM**<br>x10^3 AU/mL	**CPP-I**<br>x10^3/mL	**CPP-II**<br>x10^2/mL	**T50**<br>minutes	**CPP-II size**<br>nm	**Fetuin-A**<br>g/L
**Group mean ± SD or median (IQR)**						
Healthy Control						
Fasting	8.5<br>(7.8 - 11.0)	0.8<br>(0.4 - 1.6)	2.0<br>(1.3 - 4.3)	348 ± 43	204.1<br>(167.5 - 248.9)	0.602 ± 0.112
+30 minutes	9.7<br>(8.1 - 11.1)	1.1<br>(0.3 - 3.7)	4.7<br>(2.0 - 13.3)	371 ± 46	194.2<br>(170.7 - 261.4)	0.639 ± 0.115
+60 minutes	10.7<br>(8.4 - 13.2)	3.3<br>(1.5 - 12.0)	8.7<br>(5.3 - 12.7)	374± 50	194.0<br>(168.0 - 266.2)	0.641 ± 0.122
+120 minutes	18.3<br>(13.2 - 21.6)	5.4<br>(3.1 - 10.7)	13.0<br>(9.3 - 34.7)	370 ± 54	171.9<br>(163.0 - 225.8)	0.637 ± 0.125
+180 minutes	16.5<br>(13.5 - 23.8)	4.6<br>(2.9 - 8.4)	16.3<br>(11.3 - 41.3)	355 ± 65	199.0<br>(175.7 - 259.0)	0.637 ± 0.125
+240 minutes	14.3<br>(10.8 - 18.5)	3.7<br>(1.2 - 8.3)	10.7<br>(8.0 - 16.7)	345 ± 62	181.9<br>(164.2 - 233.4)	0.586 ± 0.112
CKD						
Fasting	21.5<br>(18.9 - 27.5)	1.2<br>(0.4 - 1.9)	1.3<br>(1.3 - 1.7)	291 ± 76	217.2<br>(180.7 - 275.1)	0.512 ± 0.199
+30 minutes	23.2<br>(18.7 - 30.8)	0.6<br>(0.2 - 1.7)	1.3<br>(1.3 - 2.7)	303 ± 74	216.4<br>(176.8 - 251.0)	0.532 ± 0.209
+60 minutes	31.9<br>(22.0 - 45.6)	4.8<br>(2.0 - 13.7)	6.3<br>(5.3 - 15.3)	324 ± 71	229.6<br>(185.4 - 246.9)	0.577 ± 0.229
+120 minutes	58.3<br>(29.6 - 68.7)	9.0<br>(4.8 - 23.3)	22.3<br>(17.3 - 32.7)	313 ± 71	213.0<br>(172.7 - 240.7)	0.538 ± 0.207
+180 minutes	49.0<br>(35.5 - 89.8)	8.9<br>(5.7 - 18.8)	17.7<br>(12.0 - 30.0)	305 ± 71	202.4<br>(157.6 - 238.0)	0.532 ± 0.214
+240 minutes	49.9<br>(33.5 - 110.3)	7.5<br>(1.9 - 14.3)	15.0<br>(9.3 - 36.7)	310 ± 75	215.3<br>(163.4 - 240.2)	0.525 ± 0.223
**Coefficient estimate (95% CI) for CKD group-by-time interaction**						
30 minutes	2.7%<br>(-11.0, 18.5)	-64.2%<br>(-87.3, 1.0)	-50.3%<br>(-77.7, 10.5)	-10.65<br>(-27.47, 6.17)	-3.5%<br>(-10.7, 4.3)	-0.02<br>(-0.05, 0.02)
60 minutes	**28.5%****<br>**(8.7, 51.7)**	9.2%<br>(-61.7, 211.7)	30.5%<br>(-40.9, 188.3)	7.71<br>(-10.83, 26.25)	-2.0%<br>(-9.9, 6.5)	0.03<br>(-0.01, 0.06)
120 minutes	6.2%<br>(-13.1, 29.8)	64.2%<br>(-44.5, 385.5)	**130.1%***<br>**(4.1, 408.6)**	7.76<br>(-13.48, 29.01)	-8.0%<br>(-16.3, 1.2)	0.01<br>(-0.02, 0.05)
180 minutes	**33.9%***<br>**(5.5, 69.9)**	81.2%<br>(-41.6, 461.9)	49.2%<br>(-32.5, 229.9)	7.67<br>(-16.54, 31.89)	**-10.9%***<br>**(-19.5, -1.3)**	0.01<br>(-0.03, 0.05)
240 minutes	**73.3%*****<br>**(30.8, 129.6)**	54.2%<br>(-53.1, 407.1)	87.0%<br>(-15.5, 313.8)	18.63<br>(-9.27, 46.52)	-2.9%<br>(-13.4, 8.9)	0.02<br>(-0.02, 0.07)

Table shows group mean ± SD or median (IQR) or linear mixed-effects model coefficient (95% CI) for group-by-time interaction. For each linear mixed-effects model, time 0 (fasting) and control group were used as the reference category for time and group, respectively. CPM, CPP-I, CPP-II and CPP-II radius were natural log transformed before fitting regression models to ensure normal distribution of residuals. For ease of interpretation coefficient estimates for log transformed variables have been exponentiated to give a percentage change from the referent category.

*p<0.05, **p<0.01, *** p<0.001.

Abbreviations: CPM, calciprotein monomers; CPP-I, primary calciprotein particles; CPP-II, secondary calciprotein particles.

Supplementary Table S3: Linear mixed-effect model and *post hoc* pairwise comparisons for serum CPP-I after re-imputation of left censored values using Tobit regression

**Timepoint**	**CPP-I (x10^3/mL)**		
Healthy Control	Group median (IQR)	Between group difference	Within group difference
Fasting	0.8 (0.4 – 1.6)	-	-
+30 minutes	1.1 (0.5 – 3.7)	-	NS
+60 minutes	3.3 (1.5 – 12.0)	-	##
+120 minutes	5.4 (3.1 – 10.7)	-	###
+180 minutes	4.6 (2.9 – 8.4)	-	###
+240 minutes	3.7 (1.2 – 8.3)	-	NS
CKD			
Fasting	1.2 (0.5 – 1.9)	NS	-
+30 minutes	0.9 (0.4 – 1.7)	NS	NS
+60 minutes	4.8 (2.0 – 13.7)	NS	##
+120 minutes	9.0 (4.8 – 23.3)	NS	###
+180 minutes	8.9 (5.7 – 18.8)	NS	###
+240 minutes	7.5 (1.9 – 14.3)	NS	##
	Between group coefficient estimate (95% CI)		
+30 minutes	-56.0% (-83.8, 19.1)		
+60 minutes	6.9% (-60.4, 188.7)		
+120 minutes	60.7% (-41.1, 338.3)		
+180 minutes	77.3% (-35.9, 390.2)		
+240 minutes	71.4% (-39.0, 381.9)		

Linear mixed-effect model and *post hoc* pairwise comparisons repeated after 8 left censored values for CPP-I were re-imputed using multi-level Tobit regression. Table shows median (IQR), linear mixed-effects model coefficient (95% CI) for group-time interaction, and *post hoc* pairwise comparisons (with Bonferroni correction for multiple comparisons). Values were natural log transformed before fitting regression models to ensure normal distribution of residuals. For ease of interpretation coefficient estimates for log transformed variables have been exponentiated to derive estimates of percentage change. *Post hoc* pairwise comparisons test for differences in mean values between groups at each timepoint, and to test for deviation from own fasting baseline within each group.

NS: $p \geq 0.05$; ##: $p < 0.01$; ###: $p < 0.001$.

Supplementary Table S4: Linear mixed-effect model and *post hoc* pairwise comparisons for serum CPP-II after re-imputation of left censored values using Tobit regression

**Timepoint**	**CPP-II (x10^2/mL)**		
Healthy Control	Group median (IQR)	Between group difference	Within group difference
Fasting	2.0 (1.5 – 4.3)	-	-
+30 minutes	4.7 (2.8 – 13.3)	-	NS
+60 minutes	8.7 (5.3 – 12.7)	-	###
+120 minutes	13.0 (9.3 – 34.7)	-	###
+180 minutes	16.3 (12.0 – 41.3)	-	###
+240 minutes	10.7 (8.0 – 16.7)	-	###
CKD			
Fasting	1.3 (1.3 – 1.7)	NS	-
+30 minutes	2.4 (2.4 – 2.7)	NS	NS
+60 minutes	8.0 (5.3 – 15.3)	NS	###
+120 minutes	22.3 (17.3 – 32.7)	NS	###
+180 minutes	17.7 (12.0 – 30.0)	NS	###
+240 minutes	15.0 (9.3 – 36.7)	NS	###
	Between group coefficient estimate (95% CI)		
+30 minutes	-35.8% (-69.7, 36.1)		
+60 minutes	59.6% (-24.2, 236.1)		
+120 minutes	**146.4%# (16.9, 419.5)**		
+180 minutes	34.9% (-36.1, 184.8)		
+240 minutes	100.2% (-5.3, 323.5)		

Linear mixed-effect model and *post hoc* pairwise comparisons repeated after 15 left censored values for CPP-II were re-imputed using multi-level Tobit regression. Table shows median (IQR), linear mixed-effects model coefficient (95% CI) for group-time interaction, and *post hoc* pairwise comparisons (with Bonferroni correction for multiple comparisons). Values were natural log transformed before fitting regression models to ensure normal distribution of residuals. For ease of interpretation coefficient estimates for log transformed variables have been exponentiated to derive estimates of percentage change. *Post hoc* pairwise comparisons test for differences in mean values between groups at each timepoint, and to test for deviation from own fasting baseline within each group.

NS: $p \geq 0.05$; #: $p<0.05$; ##: $p<0.01$; ###: $p<0.001$.

Supplementary Table S5: Linear mixed-effect model and *post hoc* pairwise comparisons for serum citrate

**Timepoint**	**Citrate (µM)**		
Healthy Control	Group median (IQR)	Between group difference	Within group difference
Fasting	77.5 (69.7 – 88.5)	-	-
+30 minutes	85.0 (76.3 – 107.1)	-	NS
+60 minutes	88.1 (82.1 – 112.5)	-	NS
CKD			
Fasting	65.4 (55.2 – 73.9)	NS	-
+30 minutes	83.0 (80.1 – 101.1)	NS	##
+60 minutes	84.5 (72.7 – 97.7)	NS	NS
	Between group coefficient estimate (95% CI)		
+30 minutes	21.0% (-6.1, 56.0)		
+60 minutes	8.7% (-16.7, 41.8)		

Table shows median (IQR), linear mixed-effects model coefficient (95% CI) for group-time interaction, and *post hoc* pairwise comparisons (with Bonferroni correction for multiple comparisons). Values were natural log transformed before fitting regression models to ensure normal distribution of residuals. For ease of interpretation coefficient estimates for log transformed variables have been exponentiated to derive estimates of percentage change. *Post hoc* pairwise comparisons test for differences in mean values between groups at each timepoint, and to test for deviation from own fasting baseline within each group.

Testing of three serves of the standard meal used showed a mean of 922.2 mg of citrate per 250mL serve.

NS: $p \geq 0.05$; ##: $p < 0.01$.

Supplementary Table S6: Linear mixed-effect models for conventional biochemical markers.

	**Phosphate** mmol/L	**Calcium** mmol/L	**Magnesium** mmol/L	**Albumin** g/L	**Bicarbonate** mmol/L
**Group mean ± SD**					
Healthy Control					
Fasting	0.96 ± 0.14	2.29 ± 0.08	0.84 ± 0.06	40 ± 2.0	27 ± 2.6
+30 minutes	0.94 ± 0.12	2.28 ± 0.07	0.83 ± 0.06	40 ± 1.8	27 ± 2.6
+60 minutes	0.91 ± 0.12	2.28 ± 0.09	0.83 ± 0.06	40 ± 2.0	27 ± 2.4
+120 minutes	0.98 ± 0.14	2.32 ± 0.11	0.83 ± 0.06	40 ± 2.2	27 ± 2.1
+180 minutes	1.03 ± 0.12	2.28 ± 0.14	0.83 ± 0.07	40 ± 2.8	27 ± 2.2
+240 minutes	1.08 ± 0.12	2.35 ± 0.11	0.84 ± 0.06	42 ± 2.6	28 ± 2.4
CKD					
Fasting	1.17 ± 0.24	2.25 ± 0.10	0.80 ± 0.08	36 ± 2.9	22 ± 3.0
+30 minutes	1.14 ± 0.25	2.24 ± 0.11	0.79 ± 0.07	36 ± 2.9	23 ± 3.7
+60 minutes	1.12 ± 0.25	2.25 ± 0.11	0.79 ± 0.07	36 ± 2.4	23 ± 3.8
+120 minutes	1.15 ± 0.24	2.27 ± 0.11	0.81 ± 0.08	37 ± 3.2	23 ± 3.6
+180 minutes	1.15 ± 0.25	2.28 ± 0.11	0.81 ± 0.08	37 ± 3.2	23 ± 3.3
+240 minutes	1.19 ± 0.25	2.29 ± 0.11	0.81 ± 0.08	37 ± 3.4	23 ± 3.4
**Between group coefficient estimate (95% CI)**					
+30 minutes	-0.01 (-0.07, 0.04)	0.00 (-0.05, 0.05)	0.00 (-0.02, 0.02)	0.16 (-0.84, 1.17)	1.13 (-0.09, 2.34)
+60 minutes	0.00 (-0.07, 0.06)	0.00 (-0.05, 0.05)	0.00 (-0.03, 0.02)	0.13 (-0.95, 1.22)	1.17 (-0.12, 2.47)
+120 minutes	-0.04 (-0.11, 0.03)	-0.01 (-0.06, 0.04)	0.01 (-0.01, 0.04)	0.34 (-0.86, 1.54)	**1.64* (0.25, 3.04)**
+180 minutes	**-0.09* (-0.17, -0.02)**	0.04 (-0.02, 0.09)	0.02 (-0.01, 0.05)	0.76 (-0.59, 2.11)	1.35 (-0.18, 2.88)
+240 minutes	**-0.10* (-0.19, -0.02)**	-0.03 (-0.09, 0.03)	0.01 (-0.02, 0.04)	-0.56 (-2.08, 0.96)	0.22 (-1.47, 1.92)

Table shows group mean ± SD or median (IQR) or linear mixed-effects model coefficient (95% CI) for group-by-time interaction. For each linear mixed-effects model, time 0 (fasting) and control group were used as the reference category for time and group respectively.

*$p<0.05$.

Supplementary Table S7: Linear mixed-effect models for PTH and iFGF23.

	**PTH** pmol/L	**iFGF23** pg/mL
**Group mean ± SD**		
Healthy Control		
Fasting	5.2 (4.6 - 6.8)	48.8 (32.9 - 68.8)
+120 minutes	4.3 (3.1 - 5.0)	40.1 (29.7 - 58.5)
+240 minutes	5.3 (4.4 - 5.9)	42.9 (32.9 - 59.8)
CKD		
Fasting	16.9 (7.1 - 31.0)	132.5 (75.1 - 238.9)
+120 minutes	15.4 (5.3 - 30.5)	114.8 (62.7 - 240.9)
+240 minutes	18.4 (5.5 - 29.3)	99.1 (72.3 - 194.6)
**Between group coefficient estimate (95% CI)**		
+120 minutes	**25.2%* (5.5, 48.5)**	7.8% (-12.9, 33.5)
+240 minutes	6.1% (-12.3, 28.3)	-7.1% (-27.9, 19.8)

Table shows group mean ± SD or median (IQR) or linear mixed-effects model coefficient (95% CI) for group-by-time interaction. For each linear mixed-effects model, time 0 (fasting) and control group were used as the reference category for time and group, respectively. PTH and iFGF23 were natural log transformed before fitting regression models to ensure normal distribution of residuals. For ease of interpretation coefficient estimates for log transformed variables have been exponentiated to give a percentage change from the referent category.

*p<0.05.

Abbreviations: PTH, intact parathyroid hormone; iFGF23, intact fibroblast growth factor-23.

Supplementary Table S8: Post-hoc pairwise comparisons for serum fetuin-A showing with and without Bonferroni correction method to adjust for multiple comparisons

Fetuin-A		Corrected (Bonferroni)		Uncorrected	
	Difference	95% CI	p-value	95% CI	p-value
**Between group**					
Diff at baseline	-0.090	-0.295, 0.116	1.000	-0.209, 0.030	0.142
Diff at 30	-0.107	-0.313, 0.100	1.000	-0.227, 0.014	0.083
Diff at 60	-0.064	-0.272, 0.144	1.000	-0.185, 0.057	0.300
Diff at 120	-0.077	-0.288, 0.133	1.000	-0.200, 0.045	0.216
Diff at 180	-0.084	-0.297, 0.129	1.000	-0.208, 0.040	0.184
Diff at 240	-0.065	-0.281, 0.151	1.000	-0.191, 0.061	0.312
**Control group**					
30 vs baseline	0.037	-0.002, 0.075	0.094	0.014, 0.059	0.001
60 vs baseline	0.039	-0.001, 0.079	0.078	0.015, 0.062	0.001
120 vs baseline	0.035	-0.008, 0.079	0.423	0.010, 0.060	0.006
180 vs baseline	0.035	-0.012, 0.082	0.810	0.008, 0.063	0.012
240 vs baseline	-0.016	-0.068, 0.036	1.000	-0.046, 0.014	0.296
**CKD group**					
30 vs baseline	0.020	-0.021, 0.061	1.000	-0.004, 0.044	0.107
60 vs baseline	0.065	0.021, 0.108	0.000	0.039, 0.090	0.000
120 vs baseline	0.048	0.000, 0.095	0.046	0.020, 0.075	0.001
180 vs baseline	0.041	-0.011, 0.092	0.488	0.011, 0.071	0.007
240 vs baseline	0.009	-0.047, 0.065	1.000	-0.024, 0.041	0.599

Post hoc pairwise comparison was performed after fitting LMM to test for differences in mean values for fetuin-A. Differences between groups (CKD v Control) at each timepoint as well as deviation from the fasting baseline within each group were examined. Table shows confidence interval and p-value with and without Bonferroni correction method to adjust for multiple comparisons.

Supplementary Table S9: Linear mixed-effect model and *post hoc* pairwise comparisons for serum CPM in those with CKD (by use of cholecalciferol)

**Timepoint**	**CPM (x10^3 AU/mL)**		
Not taking cholecalciferol	Group median (IQR)	Between group difference	Within group difference
Fasting	22.4 (13.0 - 26.4)	-	-
+30 minutes	22.2 (14.2 - 28.1)	-	NS
+60 minutes	33.7 (18.5 - 47.6)	-	###
+120 minutes	58.5 (29.6 - 79.6)	-	###
+180 minutes	57.8 (29.9 - 94.7)	-	###
+240 minutes	67.5 (25.7 - 110.6)	-	###
Taking cholecalciferol			
Fasting	20.1 (19.1 - 28.8)	NS	-
+30 minutes	25.3 (20.7 - 37.4)	NS	NS
+60 minutes	31.9 (22.5 - 45.6)	NS	NS
+120 minutes	48.9 (25.0 - 68.7)	NS	###
+180 minutes	49.0 (35.5 - 89.8)	NS	###
+240 minutes	48.7 (45.8 - 49.9)	NS	###
	Between group coefficient estimate (95% CI)		
+30 minutes	-1.2% (-20.4, 22.6)		
+60 minutes	-19.1% (-37.2, 4.2)		
+120 minutes	-17.7% (-39.5, 11.9)		
+180 minutes	-22.4% (-46.2, 11.9)		
+240 minutes	-17.2% (-46.5, 28.1)		

Linear mixed-effect model and *post hoc* pairwise comparisons of the CKD group by use of cholecalciferol (n=6). Table shows median (IQR), linear mixed-effects model coefficient (95% CI) for group-time interaction, and *post hoc* pairwise comparisons (with Bonferroni correction for multiple comparisons). Values were natural log transformed before fitting regression models to ensure normal distribution of residuals. For ease of interpretation coefficient estimates for log transformed variables have been exponentiated to derive estimates of percentage change. Model was adjusted for eGFR. *Post hoc* pairwise comparisons test for differences in mean values between groups at each timepoint, and to test for deviation from own fasting baseline within each group.

NS: $p \geq 0.05$; ###: $p < 0.001$.

Supplementary Table S10: Linear mixed-effect model and *post hoc* pairwise comparisons for serum CPP-I in those with CKD (by use of cholecalciferol)

**Timepoint**	**CPP-II (x10^3/mL)**		
Not taking cholecalciferol	Group median (IQR)	Between group difference	Within group difference
Fasting		-	-
+30 minutes	0.9 (0.3 - 2.1)	-	NS
+60 minutes	0.2 (0.1 - 0.6)	-	NS
+120 minutes	3.1 (1.7 - 7.5)	-	###
+180 minutes	6.4 (4.4 - 20.9)	-	###
+240 minutes	8.3 (4.3 - 21.6)	-	##
Taking cholecalciferol			
Fasting	1.5 (0.5 - 1.9)	NS	-
+30 minutes	1.7 (1.1 - 4.3)	NS	NS
+60 minutes	11.1 (2.5 - 16.9)	NS	#
+120 minutes	14.2 (5.1 - 24.7)	NS	###
+180 minutes	11.3 (7.8 - 18.8)	NS	##
+240 minutes	4.8 (1.9 - 8.0)	NS	NS
	Between group coefficient estimate (95% CI)		
+30 minutes	**401.7%$^{\#}$ (24.6, 1921.0)**		
+60 minutes	56.7% (-61.2, 532.1)		
+120 minutes	9.6% (-72.9, 343.4)		
+180 minutes	-11.8% (-78.3, 258.1)		
+240 minutes	-35.9% (-84.3, 161.4)		

Linear mixed-effect model and *post hoc* pairwise comparisons of the CKD group by use of cholecalciferol (n=6). Table shows median (IQR), linear mixed-effects model coefficient (95% CI) for group-time interaction, and *post hoc* pairwise comparisons (with Bonferroni correction for multiple comparisons). Values were natural log transformed before fitting regression models to ensure normal distribution of residuals. For ease of interpretation coefficient estimates for log transformed variables have been exponentiated to derive estimates of percentage change. Model was adjusted for eGFR. *Post hoc* pairwise comparisons test for differences in mean values between groups at each timepoint, and to test for deviation from own fasting baseline within each group.

NS: p≥0.05; #: p<0.05; ##: p<0.01; ###: p<0.001.

Supplementary Table S11: Linear mixed-effect model and *post hoc* pairwise comparisons for serum CPP-II in those with CKD (by use of cholecalciferol)

**Timepoint**	**CPP-II (x10^2/mL)**		
Not taking cholecalciferol	Group median (IQR)	Between group difference	Within group difference
Fasting	1.3 (1.3 - 2.5)	-	-
+30 minutes	1.3 (1.3 - 3.0)	-	##
+60 minutes	6.3 (5.0 - 12.0)	-	###
+120 minutes	22.3 (17.7 - 30.7)	-	###
+180 minutes	14.7 (10.0 - 19.3)	-	###
+240 minutes	15.3 (9.3 - 37.7)	-	###
Taking cholecalciferol			
Fasting	0.1 (0.1 - 0.1)	NS	-
+30 minutes	0.2 (0.1 - 0.3)	NS	NS
+60 minutes	0.8 (0.5 - 3.1)	NS	##
+120 minutes	2.6 (1.1 - 3.9)	NS	###
+180 minutes	2.4 (1.7 - 3.3)	NS	###
+240 minutes	1.5 (0.7 - 1.8)	NS	###
	Between group coefficient estimate (95% CI)		
+30 minutes	-4.7% (-68.1, 130.7)		
+60 minutes	3.8% (-65.4, 211.4)		
+120 minutes	-8.7% (-69.8, 175.9)		
+180 minutes	74.4% (-42.9, 432.1)		
+240 minutes	-25.4% (-75.9, 130.7)		

Linear mixed-effect model and *post hoc* pairwise comparisons of the CKD group by use of cholecalciferol (n=6). Table shows median (IQR), linear mixed-effects model coefficient (95% CI) for group-time interaction, and *post hoc* pairwise comparisons (with Bonferroni correction for multiple comparisons). Values were natural log transformed before fitting regression models to ensure normal distribution of residuals. For ease of interpretation coefficient estimates for log transformed variables have been exponentiated to derive estimates of percentage change. Model was adjusted for eGFR. *Post hoc* pairwise comparisons test for differences in mean values between groups at each timepoint, and to test for deviation from own fasting baseline within each group.

NS: $p \geq 0.05$; #: $p<0.05$; ##: $p<0.01$; ###: $p<0.001$.

Supplementary Figure S1: Pharmacokinetics of calciprotein monomers in relation to kidney function (eGFR)

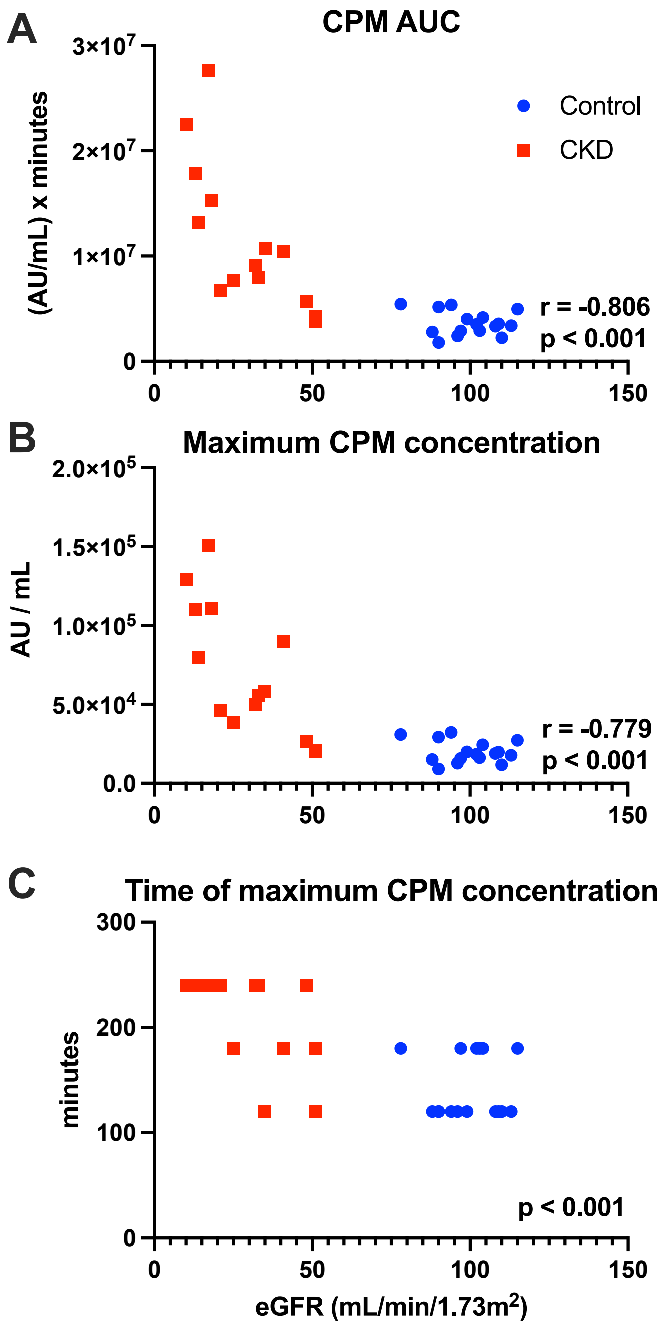

Pharmacokinetics of calciprotein monomers (CPM) in relation to kidney function (estimated glomerular filtration rate [eGFR]). (A) Area under the curve calculated using cubic spline method for time 0 until 240 minutes. (B) Maximum CPM concentration. (C) Time of maximum CPM concentration.

P-value for Pearson correlation coefficient ("r") shown for AUC and maximum concentration. P-value for time to maximum concentration calculated by chi squared test, using group and categorical time.

Supplementary Figure S2: Pharmacokinetics of CPP-I and CPP-II in relation to kidney function (eGFR)

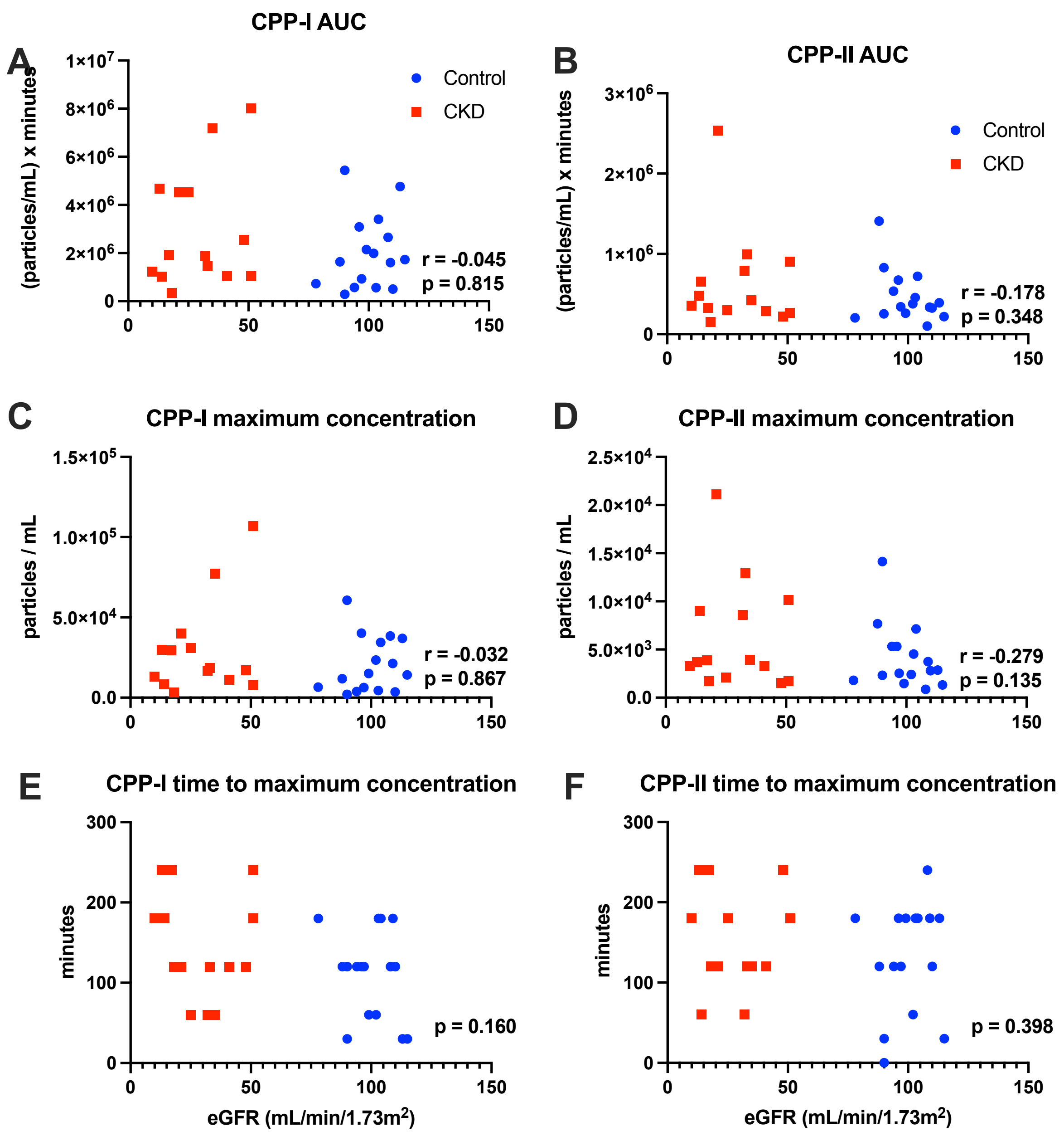

Pharmacokinetics of primary (CPP-I) and secondary (CPP-II) calciprotein particles in relation to kidney function (estimated glomerular filtration rate [eGFR]). (A & B) Area under the curve calculated using cubic spline method for time 0 until 240 minutes. (C & D) Maximum CPP-I/CPP-II concentration. (E & F) Time of maximum CPP-I/CPP-II concentration.

P-value for Pearson correlation coefficient ("r") shown for AUC and maximum concentration. P-value for time to maximum concentration calculated by chi squared test, using group and categorical time.

Supplementary Figure S3: Change in T50 and serum fetuin-A from baseline

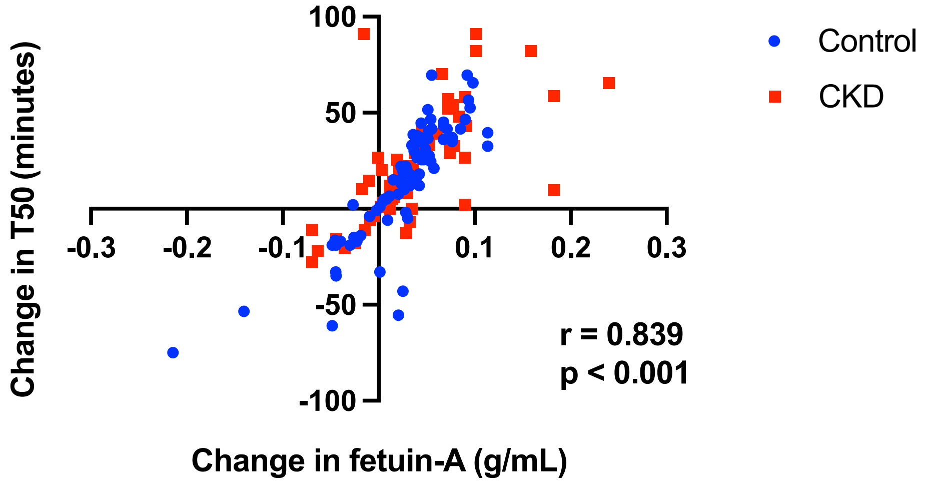

Maximum increase in T50 and serum fetuin-A from baseline.

r = Pearson correlation coefficient

Supplementary Figure S4: Change in T50 and serum citrate from baseline.

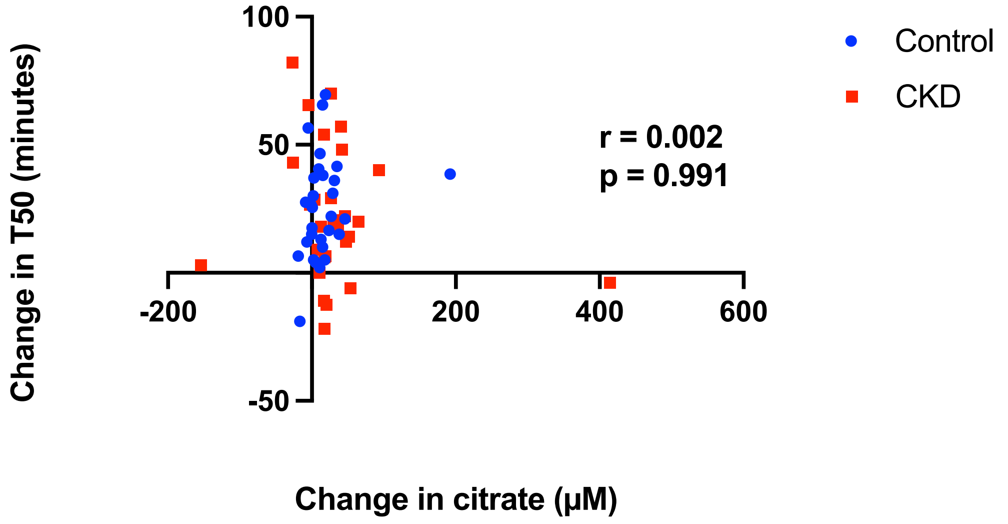

Maximum increase in T50 and serum citrate from baseline (using available data at baseline, 30 and 60 minutes).

r = Pearson correlation coefficient

Supplementary Figure S5: Change in T50 and serum bicarbonate from baseline

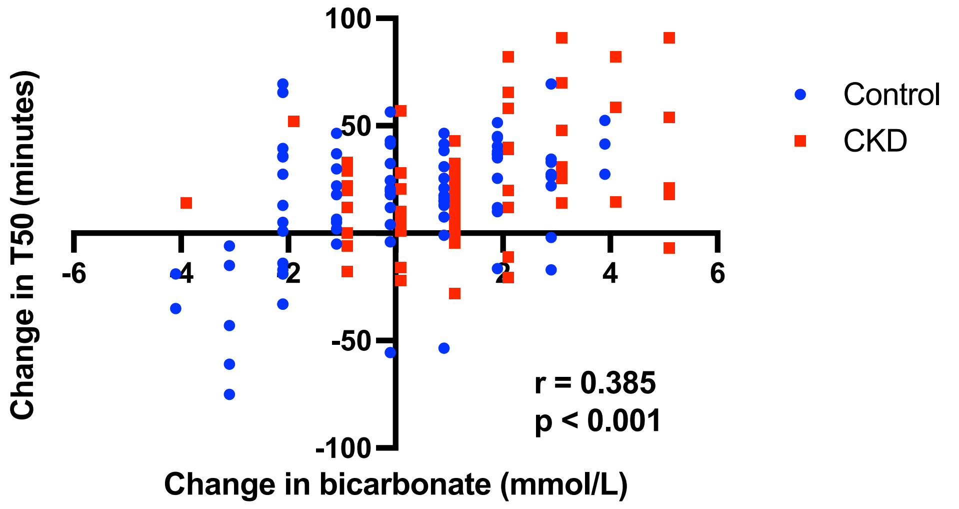

Maximum increase in T50 and serum bicarbonate from baseline.

r = Pearson correlation coefficient

Data points have been offset on the x-axis to improve clarity.

Supplementary Figure S6: Novel markers of mineral metabolism (showing individual CKD groups)

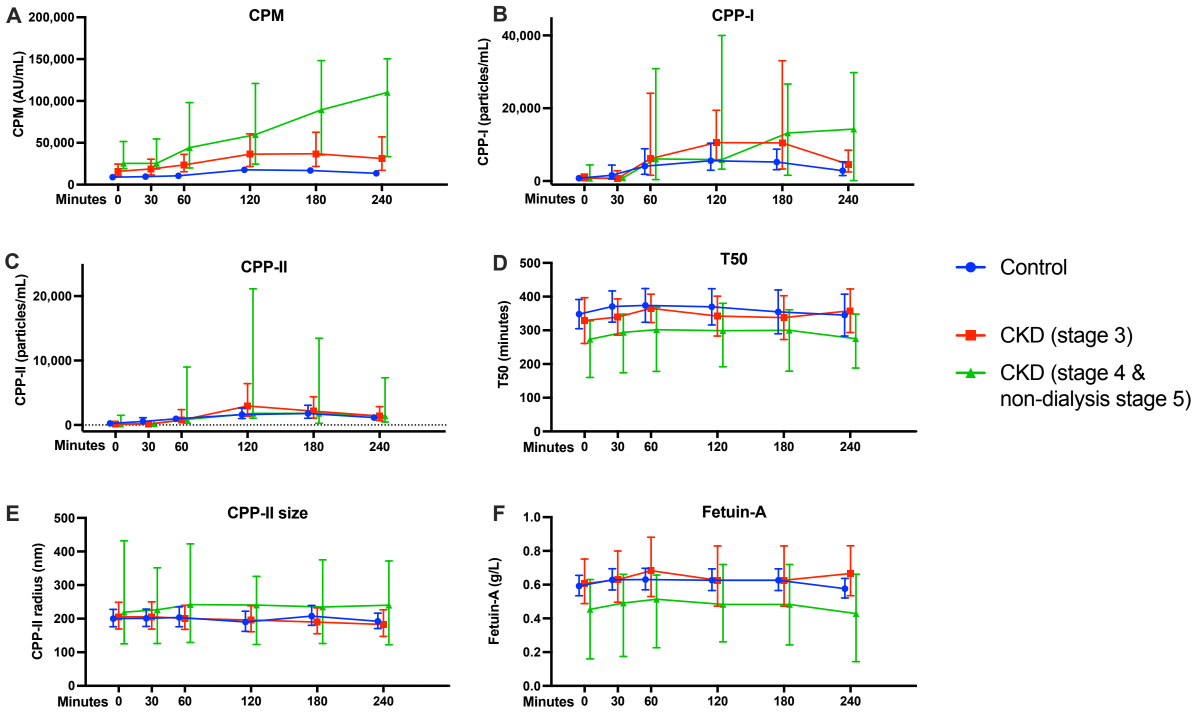

Repeated measures of novel markers of mineral metabolism depicted for control, CKD stage 3, and CKD stage 4 & non-dialysis stage 5 groups when fasting ("0") and then post consumption of a standardised meal. Data are presented as mean and 95% confidence interval or geometric mean and 95% confidence interval. Data points have been offset for clarity.

(A) Calciprotein monomers (CPM); (B) Primary calciprotein particles (CPP-I); (C) Secondary calciprotein particles (CPP-II); (D) T50; (E) Secondary calciprotein particle size (CPP-II size); (F) Fetuin-A.

Supplementary Figure S7: Novel markers of mineral metabolism (showing repeated fasting timepoints)

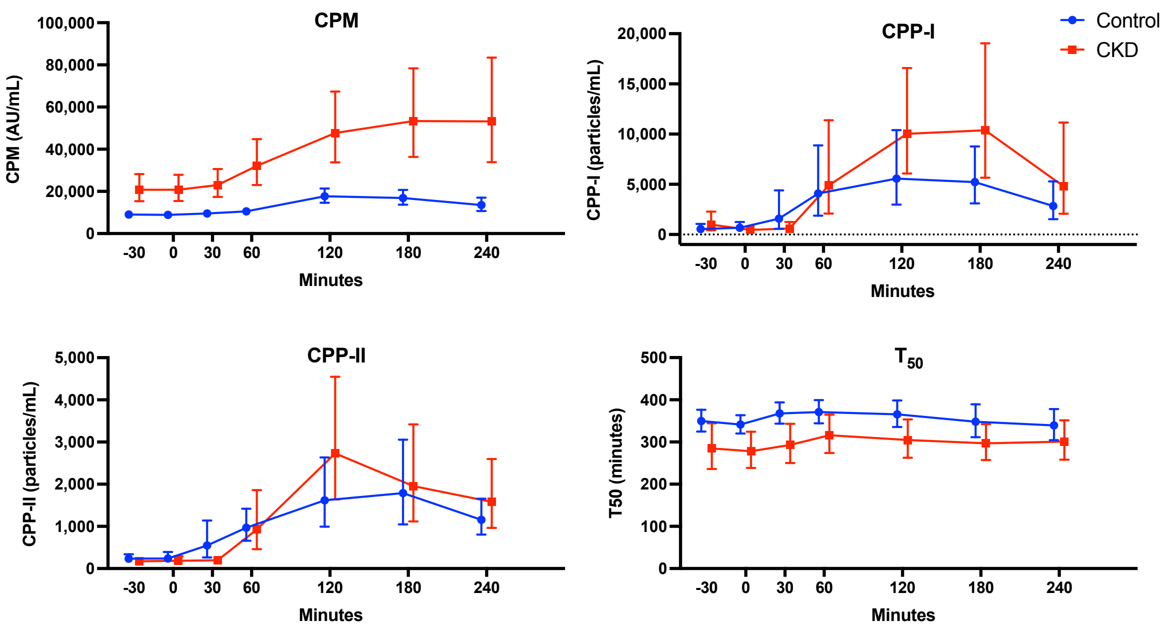

Repeated measures of novel markers of mineral metabolism for control and CKD group. Depicting repeated fasting measurements (30 minutes, and immediately prior) and post standardised meal. Data are presented as mean and 95% confidence interval or geometric mean and 95% confidence interval. Data points have been offset for clarity.

Abbreviations: CPM, calciprotein monomers; CPP-I, primary calciprotein particles; CPP-II, secondary calciprotein particles.
